# Restart errors reaction time of a two-step inhibition process account for the violation of the race model’s independence in multi-effector selective stop signal task

**DOI:** 10.3389/fnhum.2023.1106298

**Published:** 2023-02-10

**Authors:** Isabel Beatrice Marc, Valentina Giuffrida, Surabhi Ramawat, Lorenzo Fiori, Roberto Fontana, Giampiero Bardella, Sabrina Fagioli, Stefano Ferraina, Pierpaolo Pani, Emiliano Brunamonti

**Affiliations:** ^1^Department of Physiology and Pharmacology, Sapienza University, Rome, Italy; ^2^Behavioral Neuroscience PhD Program, Sapienza University, Rome, Italy; ^3^Department of Occupational and Environmental Medicine, Epidemiology and Hygiene, INAIL, Rome, Italy; ^4^Department of Education, University of Roma Tre, Rome, Italy

**Keywords:** selective stop signal task, motor inhibition, effectors coupling, race model violation, executive control

## Abstract

Goal-oriented actions often require the coordinated movement of two or more effectors. Sometimes multi-effector movements need to be adjusted according to a continuously changing environment, requiring stopping an effector without interrupting the movement of the others. This form of control has been investigated by the selective Stop Signal Task (SST), requiring the inhibition of an effector of a multicomponent action. This form of selective inhibition has been hypothesized to act through a two-step process, where a temporary global inhibition deactivating all the ongoing motor responses is followed by a restarting process that reactivates only the moving effector. When this form of inhibition takes place, the reaction time (RT) of the moving effector pays the cost of the previous global inhibition. However, it is poorly investigated if and how this cost delays the RT of the effector that was required to be stopped but was erroneously moved (Stop Error trials). Here we measure the Stop Error RT in a group of participants instructed to simultaneously rotate the wrist and lift the foot when a Go Signal occurred, and interrupt both movements (non-selective Stop version) or only one of them (selective Stop version) when a Stop Signal was presented. We presented this task in two experimental conditions to evaluate how different contexts can influence a possible proactive inhibition on the RT of the moving effector in the selective Stop versions. In one context, we provided the foreknowledge of the effector to be inhibited by presenting the same selective or non-selective Stop versions in the same block of trials. In a different context, while providing no foreknowledge of the effector(s) to be stopped, the selective and non-selective Stop versions were intermingled, and the information on the effector to be stopped was delivered at the time of the Stop Signal presentation. We detected a cost in both Correct and Error selective Stop RTs that was influenced by the different task conditions. Results are discussed within the framework of the race model related to the SST, and its relationship with a restart model developed for selective versions of this paradigm.

## 1. Introduction

The ability to inhibit an already planned response is a key component of executive control aiding the interaction with a continuously changing environment. This ability has been largely investigated by employing the Stop Signal Task (SST) (Vince, 1948; Logan and Cowan, 1984; Schall et al., 2017). The SST requires starting a movement when a Go Signal is presented (No Stop trials) and refraining from it as a Stop Signal suddenly appears in a minority of trials (Stop trials). In the typical experimental setting, participants are asked to execute or cancel simple movements such as pressing a button, moving a joystick, or reaching a peripheral visual target by an eye or an arm movement (Mirabella et al., 2006; Boucher et al., 2007; Emeric et al., 2007; Aron and Verbruggen, 2008; Brunamonti et al., 2012; Pani et al., 2014; Mione et al., 2015). Regardless of the movement taking place, a theoretical “horse race model” has long been proposed to account for the behavioral outcomes in this experimental paradigm (Logan and Cowan, 1984): a movement starts every time the Go process, triggered by the Go Signal, grows sufficiently to achieve a critical threshold. Whenever a Stop Signal occurs after the Go Signal, an independent Stop process starts racing against the Go process, and if it wins the race, it successfully interrupts the process of movement generation. One of the important aspects of the SST and the related race model is that it provides an estimate of the reactive inhibition as the time needed to stop an already planned movement Stop Signal reaction time (SSRT). The SST has been extensively employed to investigate single effector inhibition in different experimental contexts (Lavallee et al., 2014; Montanari et al., 2017; Pani et al., 2018; Andujar et al., 2022) and pathological populations (Brunamonti et al., 2011, 2014; Olivito et al., 2017; Mancini et al., 2018; di Caprio et al., 2020; Mirabella et al., 2020; Suarez et al., 2021). However, it is still an open question if the model can be easily extended to actions requiring the coordination or interaction of different body segments (Hannah and Aron, 2021; Wadsley et al., 2022). Indeed, many of our daily actions engage in the simultaneous control of more effectors (Shea et al., 2016; Hannah and Aron, 2021). For example, while driving, most of the time the hand and foot are functionally coupled to simultaneously depress the gas pedal and turn the steering wheel, but sudden events, such as the presence of an unexpected obstacle on the road, may require releasing the gas pedal while continuing to turn the steering wheel, thus stopping one effector while performing an action with the other. In several studies, the above form of selective inhibition has been investigated by employing selective versions of the SST (de Jong et al., 1995; Boucher et al., 2007; Aron and Verbruggen, 2008; Claffey et al., 2010; MacDonald et al., 2012; Xu et al., 2015; Raud et al., 2020; Wadsley et al., 2022). Typically, these tasks require reacting to a Go Signal by the simultaneous movement of two effectors (e.g., pressing a button with the left and right index fingers), and selectively cancel the movement of only one effector when a Stop Signal occurs. Analysis of muscle and neural activity during selective inhibition showed that the race model was not adequate and proposed a two-step restart model (de Jong et al., 1995; Coxon et al., 2007; Claffey et al., 2010; MacDonald et al., 2012; Wadsley et al., 2022). The restart model posits that once a Go Signal activates both effector movements, the presentation of the Selective Stop Signal consequently results in a first step of general inhibition, which dampens all the ongoing motor responses, and then a second step which restarts the movement of the effector that is not required to be stopped. In this context, in selective Stop trials, when the subjects successfully cancel the planned action of the indicated effector, the RT of the effector moving is typically longer than in No Stop trials. This elongation, called the stop interference effect (Coxon et al., 2007) is suggested as the sign of the general inhibition process. While the stop interference has been documented for correctly performed Selective Stop trials, it is not known if it affects the RT of the incorrectly moved effector in Selective Stop Error trials (Restart Errors). A stop interference effect on Stop Error trials would lengthen their RTs, accounting for a putative violation of the independence assumption of the race model. This assumption is respected when the Stop Error RT is faster than the No Stop RT and assess that the running of the Go and Stop process is not mutually interfered. The study of movement inhibition by the SST also evidenced a proactive inhibition acting on the RT of No Stop trials to face environmental changes that might occur with a known probability. In the case of a reaching or joystick movement, for example, the same movement is slower when performed in a context where a Stop Signal has a probability to occur than in a context where Stop trials are not expected (Mirabella et al., 2008; Brunamonti et al., 2014; di Caprio et al., 2020; Mirabella, 2021). Proactive inhibition during selective SST has been observed in contexts where information about the effector to be inhibited was provided in advance. The foreknowledge of the effector to be inhibited reduced the stop interference effect and delayed the SSRT, compared to conditions in which such information was not provided (Aron and Verbruggen, 2008; Raud and Huster, 2017; Cirillo et al., 2018). In the present work, we investigated if the stop interference increased the RTs of Stop Error trials of a selective SST and if foreknowledge of the effector to be inhibited influences this interference.

To this end, we developed a multi-effector selective SST requiring the participants to respond to a Go Signal by simultaneously extending their wrist and flexing their foot. In different stopping conditions, participants had to stop the movement of both effectors (non-selective Stop version) or selectively inhibit the movement of only one of them (selective Stop version). Non-selective Stop versions of the task were used as a control for the selective Stop versions. Proactive inhibition was manipulated by presenting the different conditions of the task in blocks of trials always requiring the inhibition of the same effector(s) or in a Mix condition, in which the information of the effector(s) to be stopped was provided at the time of the Stop Signal presentation. The two modalities of presentation of the task were two different contexts where the foreknowledge of the effector to be inhibited was provided or not. To measure the beginning of motor activity, here we used the onset of EMG activity at the level of the effector muscles that produces a response. This variable can be recorded in any task that requires overt responses, as well as in tasks that require more complex movements that activate different muscle groups (Tao et al., 2018).

## 2. Materials and methods

### 2.1. Participants

We estimated the sample size on the basis of a power of 0.9 to detect an effect size in a within and between subject design of 0.55, using GPower 3.1.9 (Faul et al., 2007, 2009), based on a previous study that employed a similar task and sample size (Coxon et al., 2007). This estimate corresponded to 9. Nine participants (2 females and 7 males) aged between (25 and 30 years old; mean = 27; SD = 2) were recruited for the study. All participants were right-handed, according to the Edinburgh Handedness Inventory (Oldfield, 1971), and had normal or corrected to normal vision. Participants performed the different versions of the multi-effector selective SST on different days. The order of presentation of the different blocks was randomized across subjects. All procedures were performed in accordance with the Declaration of Helsinki and after obtaining written informed consent from each participant. The procedure was approved by the Ethics Committee of “Roma Tre” University.

### 2.2. Multi–Effector selective SST

**FIGURE 1:**
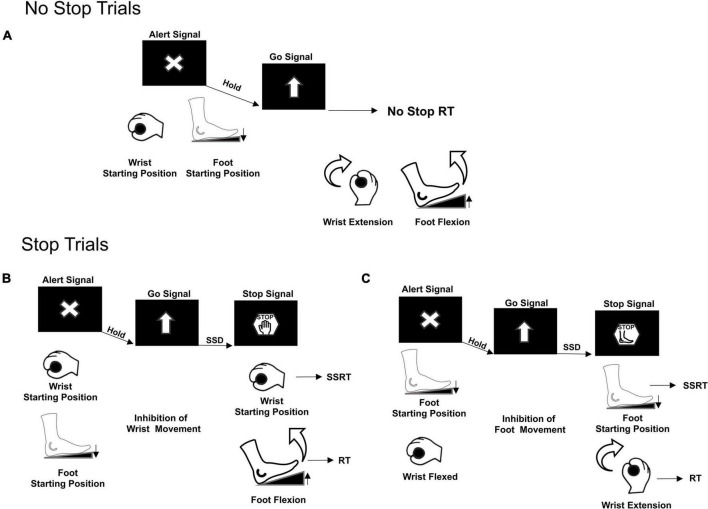
Multi-Effector selective SST. Two types of trials were presented, No Stop trials **(A)** and Stop Trials **(B,C)**. Movement of both effectors, wrist and foot, was required only in No Stop trials. Movement cancelation was required only in Stop trials. Depending on the different Stop versions of the task, the appearance of the Stop Signal informed either to cancel the movement of both effectors, Stop Both not shown, or to selectively cancel either the wrist movement, but still move the foot **(B)** or the foot movement, but still move the wrist **(C)**. RT, Reaction Time; SSD, Stop Signal Delay; SSRT, Stop Signal Reaction Time.

Participants were seated on a chair 60 cm away in front of a computer monitor with a black background. Their right arm was laying on a table as they handled a bar and pushed their right foot on a pedal. Each trial started when both the effectors reached their starting position: the wrist flexed toward the left and the foot pedal pushed (Figure 1A). The correct starting position was signaled by two electrical switches that triggered the appearance of an Alert Signal on the screen.

**FIGURE 2:**
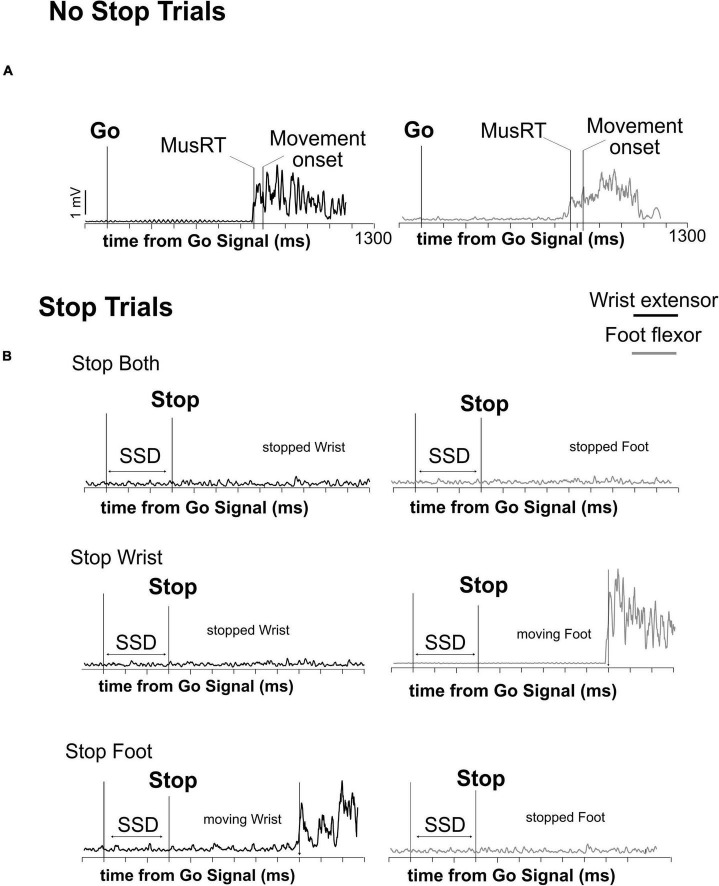
Time evolution of muscular activity (EMG) in the different trial types. Activation’s profile of agonist muscles activity of wrist and foot in a single correct No Stop trial **(A)** and Stop trials **(B)** during the execution of the three versions of the multi-effector selective SST. The onset of muscle activity (MusRT) and the behavioral response (Movement onset) are indicated in panel **(A)**. In panel **(B)** only MusRT is indicated.

Following a variable holding time (800–1000 ms), a Go Signal (upward pointing arrow), that required participants to simultaneously perform a wrist and foot movement as fast as possible, was presented. In 70% of the trials (No Stop trials, Figure 1A), subjects had to move the wrist and the foot within 1300 ms (upper RT limit) and with a delay between them of a maximum 200 ms, otherwise the trial was aborted. In 30% of the trials (Stop trials) a Stop Signal was presented after a variable delay (Stop Signal Delay, SSD) from the Go Signal. The Stop Signal could instruct the participants to cancel the movements for both effectors (Stop Both version, not shown) or to selectively stop the movement either of the wrist (Stop Wrist version, Figure 1B–left panel) while allowing to move the foot, or the foot (Stop Foot version, Figure 1C–right panel) while moving the wrist. Each version of the Stop trials was signaled by a specific Stop Signal. For the Stop Both version, it was an empty red octagon. For the Stop Wrist version and the Stop Foot version, it was a red octagon surrounding the image of a hand or a foot, respectively (Figure 1B). In Stop Both trials, participants had to keep both effectors in the starting position for at least 1300 ms after the Stop Signal to perform a Stop Correct trial; conversely, if one or both effectors moved, the trial was a Stop Error trial. In the Stop Wrist version, to perform a Stop Correct trial, they had to maintain the wrist in the starting position and to move the foot within the upper RT limit. Stop trials in which the Wrist moved were Stop Error trials. In the Stop Foot version, Stop trials that ended with the foot stationary on the pedal and the wrist moved before reaching the upper RT were Stop Correct trials, whereas the trials in which the foot released the starting position were Stop Error trials. Trials performed correctly and those performed incorrectly were signaled by two different acoustic feedbacks. During the performance of the task, the activity of two muscles (Figure 2), respectively acting as an agonist for the wrist extension (*extensor carpi ulnaris*) and as an agonist for the foot flexion (*tibialis anterior*), was recorded by a surface EMG device. EMG signals were recorded at a frequency of 6104 Hz, by the signal acquisition device, in accordance with European Surface Electromyography Recommendations (Hermens et al., 2000) the Atlas of Muscle Innervation Zones (Barbero et al., 2012), and best practices (Merletti and Muceli, 2019; Merletti and Cerone, 2020).

### 2.3. Experimental procedure

Participants executed the three different Stop versions in two different Task conditions: Block and Mix. In the Block condition each version of the Stops, namely Stop Both, Stop Wrist or Stop Foot, was presented block wise. In contrast, in the Mix condition, the three versions of Stops were intermingled. Therefore, in the Block condition, subjects knew in advance which effector was to be stopped; conversely, in the Mix condition, the participants knew which effector was to be stopped only when the specific Stop Signal was presented. In this condition, 1/3 of Stop trials were Stop Both, 1/3 were Stop Wrist, and 1/3 were Stop Foot trials. In Stop trials, an adaptive algorithm adjusted the SSDs based on the performance: starting from an initial value of 50 ms, SSDs following Stop Correct trials were increased by 50 ms while SSDs following Stop Error trials were decreased by 50 ms. In the Mix condition, three independent adaptive algorithms adjusted the SSD for the Stop Both, Stop Wrist, and Stop Foot versions. The SSD starting value and step were selected for each participant and Stop version depending on the RT observed in preliminary blocks of familiarization with the task. To avoid slowing down of the responses, participants were instructed to respond as fast as possible to the Go Signal. Data acquisition for each participant was completed in 2 days, one for the Block condition and the other for the Mix condition. The order of presentation of the Block and Mix conditions was randomized among subjects, as well as the order of presentation of the three versions of the Stop task within the Block condition. Each participant completed 300 trials for each of the different Stop versions presented in the Block condition, and a total of 600 trials in the Mix condition, divided into 3 separate chunks of 200 trials. Participants performed a training session of about 100 trials in the Mix condition before starting the data acquisition.

### 2.4. Data processing and analysis

Electromyographic signals (EMG) of wrist and foot agonist muscles obtained in each trial were first rectified and then smoothed by a moving average. We used this processed signal to compute the latency of the muscle onset activity (Muscle reaction time; MusRT), i.e., the time point from the Go Signal at which the signal exceeded by at least 2.5 standard deviations of the average muscle activity during the 100 ms preceding the Go Signal presentation (Figures 2A, B). In the same trials, we computed the RT as the time between the Go Signal and the release of the electrical contact. We computed the time between the MusRT and the onset of movement and compared them between the Stop versions and among Task conditions to assess that this time was not influenced by the context. Then we used the MusRT as a measure of the effector activation occurring earlier in the cascade of events leading to the effector movement. MusRTs were compared across the No Stop and Stop trials with different Stop versions during the Block and the Mix conditions to evaluate: (1) if there is a difference between the No Stop MusRT observed in the Block and Mix conditions, highlighting a proactive inhibition; (2) if the presentation of the Go Signal activated a common Go process that triggered both the wrist and the foot movements, or if it started two independent Go processes for the two effectors; (3) if the stop interference on both Correct and Error Stop trials was influenced by a proactive inhibition; and finally, (4) if in the selective Stop versions, the stop interference lengthened the Stop Error MusRT, and if that was the case, was it responsible for violation of the independence between the Stop and Go processes. To answer the first two questions, we compared the average MusRTs between effectors and across the different experimental conditions. For testing the assumption of independence of the race model, we tested if the Stop Error MusRTs were shorter than the average No Stop MusRTs in each Stop version (Stop Both, Stop Wrist, and Stop Foot) and Task condition (Block and Mix). To evaluate the stop interference, we compared the MusRTs of the moving effector in the Stop Wrist and Stop Foot Correct trials with the MusRTs of the corresponding No Stop trials. Data processing and analysis were performed by custom functions developed in MATLAB.^1^ Comparisons between Stop versions and Task conditions were performed by one-way or factorial ANOVAs, followed by the *post hoc* comparisons by Bonferroni test. *T*-tests were used to compare the distributions of MusRTs in No Stop and Stop Error trials. Reference values for effect size measurements, yet η*_*p*_^2^*, are as follows: 0.01–small effect size; 0.06–medium effect size; 0.14 or higher–large effect size.

## 3. Results

### 3.1. Muscular response times and temporal coupling between effectors do not change across task conditions in No Stop trials

Participants performed No Stop trials by initiating a movement with both effectors after the Go Signal. Here we first tested if the different task contexts (Block or Mix condition) influenced the delay between the muscle activity onset (MusRT) and the movement onset (RT), yet the release of the starting position of both the wrist and the foot movement. The average values across participants in each task condition are reported in Table 1.

**TABLE 1:** Average delay (Mean and ± 1 SD) between muscle activation (MusRT) and onset of movement in No Stop trials reaction time (RT) and average MusRTs for wrist and foot movements.

Task condition	Wrist MusRT	Wrist delay (RT-MusRT)	Foot MusRT	Foot delay (RT-MusRT)
Stop Both	445 (± 97) ms	89 (± 28) ms	482 (± 72) ms	141 (± 25) ms
Stop Wrist	472 (± 95) ms	98 (± 28) ms	478 (± 96) ms	139 (± 34) ms
Stop Foot	451 (± 132) ms	90 (± 26) ms	515 (± 92) ms	132 (± 35) ms
Mix	472 (± 100) ms	92 (± 22) ms	514 (± 83) ms	141 (± 24) ms

A two-way repeated measures ANOVA with factors Effector (Wrist; Foot) and Task conditions (Stop Both; Stop Wrist; Stop Foot; Mix) revealed a significantly longer delay in foot movements than in wrist movements, *F*_(1,8)_ = 74.54; *p* < 0.001, η*_*p*_^2^* 0.90, while no main effect of the Task conditions was observed, *F*_(3,24)_ = 0.29; *p* = 0.83, η*_*p*_^2^* 0.03, nor a significant interaction was detected, *F*_(3,24)_ = 0.92; *p* = 0.45, η*_*p*_^2^* 0.10. These data revealed that the delay was constant and unaffected by Stop versions or task context. Therefore, we employed the MusRTs as a measure of movement onset of both the wrist and the foot in each task condition (Table 1). We used a two-way repeated measures ANOVA with factors Effector (Wrist; Foot) and Stop version (Stop both; Stop Wrist; Stop Foot; Mix), to test if the MusRTs of both effectors were influenced by the task context and observed that the foot MusRTs was significantly longer than the wrist MusRTs, *F*_(1,8)_ = 9.03; *p* = 0.01, η*_*p*_^2^* 0.53. No significant main effect of the Task condition was detected, *F*_(3,24)_ = 0.35; *p* = 0.78, η*_*p*_^2^* 0.04; while a significant interaction was observed between the two factors, *F*_(3,24)_ = 6,21; *p* = 0.002, η*_*p*_^2^* 0.43. However, the Bonferroni *post hoc* comparisons revealed that the MusRTs of both the wrist and the foot were not different across the different Stop versions (all *ps* > 0.05). The observation of an average delay of 46 ms across Stop versions suggests the presence of a temporal coupling between effectors while performing the No Stop trials. This evidence was further supported by significant correlation between wrist and foot MusRTs in the No Stop trials for each participant in the different experimental conditions (Spearman correlation: *r* = 0.8*; SD* ± 0.08; all *ps* < 0.05) and a lack of differences between the different Task conditions, *F*_(1,32)_ = 0.22; *p* = 0.85. Overall, these results indicate that the presentation of the Go Signal activated a common Go process that triggered both wrist and foot movements. Furthermore, they show that Task conditions did not affect the response time to the Go Signal, ruling out the effect of the context on these trials.

### 3.2. Estimate of stopping interference effect on the moving effector

**FIGURE 3:**
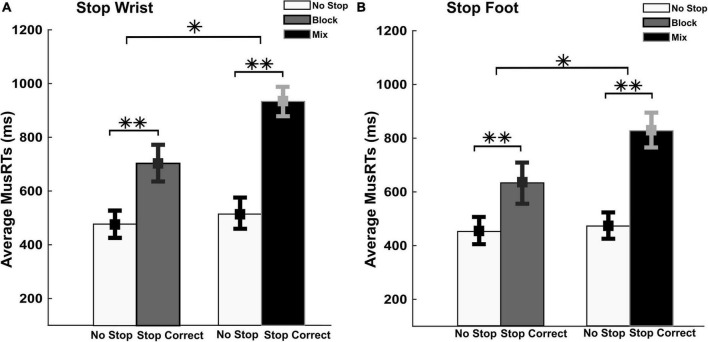
Evaluation of the moving effector and the stopping interference in Stop Correct trials. No Stop and Stop Correct MusRTs in both, selective Stop Wrist **(A)** and foot **(B)** in Block and Mix task condition. ^**^p < 0.001 and *p < 0.05.

Here we quantified the amount of stop interference in the two task contexts. To this aim, we assessed whether, during Stop Correct trials, the MusRTs of the moving effectors during selective Stop Correct Trials (wrist or foot) were delayed compared to No Stop MusRTs (Aron and Verbruggen, 2008; Bissett and Logan, 2011; Wadsley et al., 2022). Figure 3 displays the averages of the Stop Correct and No Stop MusRTs for both wrist and foot movements during selective inhibition.

A two-way repeated measures ANOVA with factors Trial Type (No Stop; Stop Correct) and Task Conditions (Block; Mix) was performed on MusRTs. In Stop Wrist (Figure 3A), the analysis revealed a significant main effect of Trials Types, *F*_(1,8)_ = 210.26; *p* < 0.001, η*_*p*_^2^* 0.96, displaying significantly longer Stop Correct MusRTs (Means, Block = 704 ms; Mix = 933 ms) than No Stop MusRTs (Means, Block = 478 ms; Mix = 514 ms). The analysis also revealed a main effect of the Task Condition, *F*_(1,8)_ = 11.00; *p* = 0.01; η*_*p*_^2^* 0.58, indicating that the MusRTs in the Mix condition were longer than in Block condition. A significant interaction between the two factors was also detected, *F*_(1,8)_ = 25.34; *p* = 0.001; η*_*p*_^2^* 0.76, revealing that the increase in MusRTs during Stop Correct trials was higher in Mix than in Block task condition (Bonferroni *post hoc*: *p* < 0.05). Similarly in Stop Foot condition (Figure 3B), the analysis revealed a significant difference between the two trial types, *F*_(1,8)_ = 96.44; *p* < 0.001, η*_*p*_^2^* 0.92; with significantly longer Stop Correct MusRTs (Means, Block = 632 ms; Mix = 826 ms) than No Stop MusRTs (Mean, Block = 451 ms; Mix = 472 ms); also a main effect of the Task Condition, *F*_(1,8)_ = 7.68; *p* = 0.02; η*_*p*_^2^* 0.49 and a significant interaction between the two factors was detected, *F*_(1,8)_ = 6.41; *p* = 0.03; η*_*p*_^2^* 0.44.

These data show that when a selective inhibition is required the moving effector is slowed down. These findings support the idea that in the Mix conditions, the higher cognitive demands generated a stronger interference of the Stop process on the moving effector that is reflected on Stop Correct MusRTs.

### 3.3. Test of independence assumption and SSRT estimates

**FIGURE 4:**
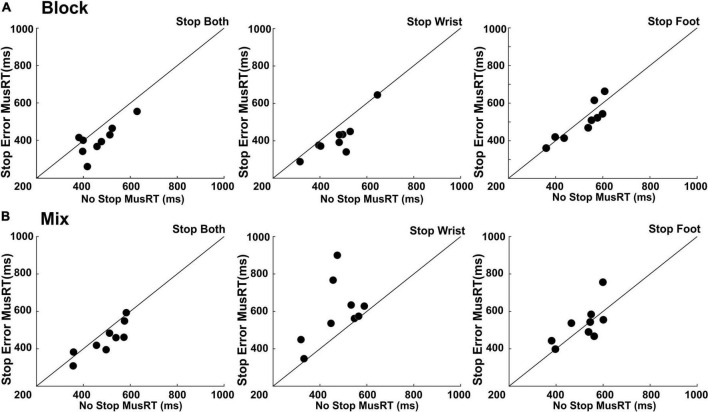
Test of independence assumption. Scatter plots of Stop Error MusRTs respect that of No Stop trials in the different selective stop conditions presented in Block **(A)** and Mix **(B)** design. Each dot represents a participant. Equality line is also depicted.

We then tested whether the collected data satisfied the independence assumption in each Task condition and each Stop version by verifying, through paired *t*-tests, that the Stop Error MusRTs were faster than the average MusRTs in No Stop trials (Figure 4). We observed that this requirement was respected only in specific Task conditions (Figure 4). Figures 4A, B (left panels) display that across participants, the independence between the Go and Stop process was accomplished in Stop trials, hence not requiring any effector selection in the Stop Both version.

In both Block and Mix conditions of Stop Both version, Stop Errors where on average faster than No Stop trials: (Block condition: Stop Error MusRTs (*M* = 405 ms, SD = 81 ms; No Stop MusRTs (*M* = 464 ms, SD ± 82 ms), *t*(8) = 3.30, *p* = 0.01); Mix condition; Stop Error MusRTs (*M* = 450 ms, SD ± 87 ms); MusRTs (*M* = 493 ms, SD ± 90 ms); *t*(8) = 2.63, *p* = 0.02). The same pattern was found in Block condition of Stop Wrist (Figure 4A, central panel), with Stop Error (*M* = 414 ms; SD ± 100 ms) being shorter than No Stop (*M* = 472 ms; SD ± 95 ms), *t*(8) = 3.41, *p* = 0.008, and in the Block condition of Stop Foot (Figure 4A, right panel), where on average Stop Error MusRTs (*M* = 502 ms; SD = ± 98 ms) were shorter than No Stop MusRTs (*M* = 515 ms; SD ± 92 ms), although in this case the numerical difference did not reach statistical significance, *t*(8) = 0.81, *p* = 0.43. In none of the Mix conditions requiring selective inhibition, i.e., Stop Wrist and Stop Foot, the independence assumption was accomplished. In Stop Wrist (Figure 4B, central panel), the average Stop Error MusRTs was slower (*M* = 600 ms; SD ± 163 ms) than in No Stop Trials (*M* = 472 ms; SD ± 100 ms), *t*(8) = −2.57, *p* = 0.03. A similar result was obtained in Stop Foot trials (Figure 4B, right panel), where Stop Error MusRTs (*M* = 530 ms; SD ± 103 ms) were slower than No Stop MusRTs (*M* = 514; SD ± 83 ms), *t*(8) = −0.64, *p* = 0.53. To summarize, a clear violation of the assumption of independence occurred when a selective inhibition was required in the Mix condition (Figure 4B, central and right panels), a task condition where participants had no anticipation of which effector should be inhibited until the Stop Signal was presented. In all the task conditions in which the independence assumption was respected, we estimated the SSRT to investigate if the different contexts, yet task conditions, influenced the time evolution of the inhibition process (Table 2).

**TABLE 2:** Mean Stop Signal reaction time (SSRTs) and SDs across participants in task conditions where the assumption of independence was respected.

Task condition	Block	Mix
Stop Both	162 (± 51) ms	140 (± 40) ms
Stop Wrist	155 (± 60) ms	Not respected
Stop Foot	141 (± 65) ms	Not respected

A one-way ANOVA comparing Stop Both Block, Stop Wrist Block, Stop Foot Block and Stop Both Mix conditions did not detect significant differences between the SSRT, *F*_(1,30)_ = 0.84; *p* = 0.48, η*_*p*_^2^* 0.07. These data suggest that a similar inhibitory process took place in the conditions with anticipation of selective inhibition (Block) or when both effectors had to be stopped (Stop Both). On the contrary, conclusions on selective inhibition could not be drawn for Mix condition since the violation of independence did not allow a reliable estimate of the SSRT.

### 3.4. Evaluation of Stop Errors as Compliant and Non-Compliant with the race model assumption of independence

**FIGURE 5:**
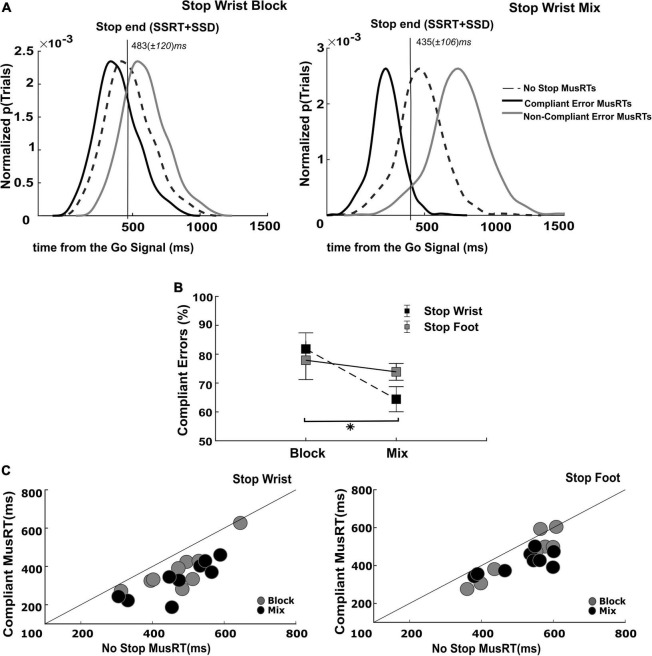
Evaluation of assumption of context independence in Compliant Stop Error trials. **(A)** Identification of Compliant and Non-Compliant Stop Error MusRTs; **(B)** proportion of Compliant Error trials in each Stop version and task condition (*p < 0.05); error bars indicate S.E.M. **(C)** Testing of assumption of independence in Compliant Stop Error trials.

The previous analysis revealed that in a portion of the Stop trials of the Mix condition, the Stop Both trials, the assumption of independence was respected while it was not in the two types of selective Stop trials. In this task condition, the selective and non-selective Stop trials were randomly intermingled with no possibility of predicting the Stop version until the presentation of the Stop Signal. The lack of foreknowledge of the effector(s) to be inhibited in this condition favored a strategy where a global inhibition acted on all the effectors, followed by the restarting of the effector required to keep moving. In this condition we expected a proportion of Stop Error trials occurring in the restart phase (second step), with the MusRT paying the cost of the previous general inhibition (stopping interference), and hence being longer than those occurring during the previous phase (first step). To gain more insight on the dynamics of this process, we analyzed the length of the Stop process in Stop Errors trials by quantifying the proportion of those occurring after the completion of the first inhibition step. Here, we estimated this time point relative to the Go Signal (Figure 5A) by relying on the SSRTs obtained by the non-selective Stop trials and the average SSDs of the selective conditions (Stop End: SSRT + SSD).

The portion of Stop Error MusRTs faster than the Stop End was Compliant with the race model’s assumption, while the slower portion of Stop Error MusRTs, completing after the Stop End, was Non-Compliant. The same analysis was applied to the selective Stop Error MusRTs in the Block condition, by using the SSRT of the Stop Both version to estimate the end of the Stop process. We selected for each participant the proportion and the average value of Compliant and Non-Compliant Stop Errors in the selective Stop versions of the task of the Block and Mix condition. Figure 5A displays across all participants and trials the distribution of Compliant (black lines) and Non-Compliant (gray line) Stop Error MusRTs during the Stop Wrist task in Block (left panel) and Mix condition (right panel), together with No Stop MusRTs (dashed line). As expected, we observed that the distribution of Compliant Stop Errors was shifted to the left of the No Stop MusRTs, while that of Non-Compliant Stop Errors was shifted to its right. Comparable distributions were obtained for the Stop Foot condition (not shown). Z-tests were used to compare the different Compliant Stop Errors distributions across conditions (Figure 5B). They revealed that the proportion of Compliant Stop Errors was lower in the Mix condition than in Block, with a significant reduction in Stop Wrist (Block: *M* = 82%; Mix: *M* = 64%), z = 2.86, *p* < 0.001; but not in Stop Foot (Block: *M* = 77%; Mix: *M* = 74%), z = 0.31, *p* > 0.05; thus, confirming that in the Mix condition for the Stop Wrist, a higher number of Stop Errors was affected by the lack of anticipation of which effector should be inhibited. Further, we confirmed that the Compliant Stop Errors respected the independence assumption by verifying that their MusRTs to be significantly faster than those of No Stop trials. Figure 5C displays that this requirement was respected in both Mix and Block conditions, Stop Wrist Block (*M* = 380 ms; *SD* ± 109 ms), *t*(8) = 4.58, *p* < 0.001; Stop Wrist Mix (*M* = 332 ms; *SD* ± 96 ms), *t*(8) = 7.04, *p* < 0.001; Stop Foot Block (*M* = 454 ms; *SD* ± 115 ms), *t*(8) = 4.12, *p* = 0.003; Stop Foot Mix (*M* = 417 ms; *SD* ± 55 ms), *t*(8) = 5.14, *p* < 0.001. We further confirmed the role of the task context in determining the longest Stop Errors MusRTs in the Mix conditions by performing a two-way repeated measures ANOVA with factors Selective Stop Versions (Stop Wrist; Stop Foot) and Task Conditions (Block; Mix). In fact, we found that overall the Stop Errors in the Mix condition (Stop Wrist: z-scored *M* = 0.98; *SD* ± 0.98; Stop Foot: z-scored *M* = 1.11; *SD* ± 2.30) were longer than in the Block condition (Stop Wrist: z-scored *M* = −0.36; *SD* ± 0.27; Stop Foot: z-scored *M* = −0.41; *SD* ± 0.33), concurring to a significant effect of the Task condition, *F*_(1,8)_ = 11.14; *p* = 0.01, η*_*p*_^2^* 0.58; however, no differences were found between Stop Wrist and Stop Foot, *F*_(1,8)_ = 0.01; *p* = 0.895, η*_*p*_^2^* 0.002; furthermore no significant interaction between the factors was detected *F*_(1,8)_ = 0.10; *p* = 0.75, η*_*p*_^2^* 0.01.

A previous work (Bissett et al., 2021) showed that the violation of independence occurs mainly for the shorter SSDs. Here, we tested if this was also the case with our data by computing the average SSD of Compliant and Non-Compliant Error in each condition of the task and Selective Stop Versions (Table 3). A two-way ANOVA with factors Stop Error (Compliant; Non-Compliant) and Task Conditions (Block; Mix) revealed that the SSDs for Compliant Stop Error trials were on average higher than they were for Non-Compliant Stop Error trials, *F*_(1,7)_ = 15.55; *p* = 0.005, η*_*p*_^2^* 0.69, with no effect of the Task condition, *F*_(1,7)_ = 1.40; *p* = 0.29, η*_*p*_^2^* 0.16. No interaction between the factors was detected, *F*_(1,7)_ = 0.02; *p* = 0.81, η*_*p*_^2^* 0.002.

**TABLE 3:** Average Stop Signal Delay (SSDs) of both Compliant and Non-Compliant Stop Error trials.

Conditions	Block	Mix
Stop Wrist	Compliant 358 (± 118) ms	311 (± *107*) ms
	Non-Compliant 279 (± 138) ms	278 (± *110*) ms
Stop Foot	Compliant 402 (± 122) ms	368 (± *81*) ms
	Non-Compliant 366 (± 123) ms	285 (± *108*) ms

Block and Mix task conditions SSDs averaged across participants.

Overall, these results revealed that in selective Stop trials a proportion of Stop Error trials was not compliant with the race model and that the proportion of Non-Compliant error trials was higher and with longer MusRTs in the Mix condition. If the Non-Compliant trials were kept in the distribution of Stop Error MusRTs, the test of independence between the Go and the Stop process failed. However, once isolated from the Compliant MusRTs, the assumption of the race model can be used for estimating the SSRT. A one-way ANOVA comparing Selective Stop Versions among Block and Mix conditions did not detect significant differences between the SSRTs, *F*_(5,45)_ = 0.36; *p* = 0.86, η*_*p*_^2^* 0.03. Here we observed that the estimated SSRT was not statistically different from that of the non-selective condition.

## 4. Discussion

We studied the selective inhibition between two effectors (i.e., wrist and foot) during a task in which participants were instructed to respond to a Go Signal with a simultaneous movement of both the effectors. Selective inhibition was tested under two different contexts, with and without prior knowledge of the effector to be stopped. The two task conditions (i.e., Block and Mix) corresponded to these contexts where the same task was performed under different levels of difficulty. As a control condition, a version of the task not requiring selective inhibition was presented. The analysis of inhibitory performance across the different experimental conditions fitted the prediction of a two step restart model of inhibition, where the level of difficulty modulated the degree of stop interference on both the Correct and Error Stop trials.

### 4.1. A common Go process subtended the movement of both effectors in the different experimental conditions

The basic task required the participants to respond to the Go Signal starting the movement of both the wrist and the foot. Since the two movements were paired in each No Stop trial of all the different Task conditions, we first asked if the movement of the two effectors was triggered by a single Go process or not. Once verified that the Task condition did not influence the delay between the muscle activation and the effector movement onset (Table 1), we focused our analyses on the muscle activation time, the MusRTs, as a signature of the completion of the Go process. Our analysis detected that the MusRT of the same effector was comparable across the different Task and Stop conditions, while the foot MusRT was slower than that of the wrist (Table 1). The significant correlation between the wrist and foot MusRTs obtained from the same No Stop trials for each participant and task condition, as well as across subjects suggested that, even though the onset of wrist muscle preceded the foot muscle, this delay was unlikely due to two different ongoing Go processes. Similar analyses performed for coordinated eye and hand movements have revealed that the difference between the two effectors was related to the different physical characteristics of the different effectors, rather than different subtending Go processes (Gopal et al., 2015). Coherently, we interpreted the observed average delay of 46 ms in MusRTs as consistent with the assumption that the initiation of a movement of a greater mass takes longer than a smaller mass (Greg Anson, 1982). Overall, these results are consistent with the hypothesis of a functional coupling between the wrist and foot movement induced by the task conditions (Shea et al., 2016).

### 4.2. Violation of race model independence in selective but not in non-selective stop conditions

Since we conform to the hypothesis of the functional coupling between the two effectors in No Stop trials, when selective inhibition is required, the uncoupling of the two effectors would allow one of them to complete the movement. Within the framework of the restart model, the decoupling should follow the general inhibition that pauses the ongoing Go process. The analysis of the selective Stop Correct MusRTs suggests that the effort in decoupling the two effectors for completing the proper movements is affected by the difficulty of the task condition. Figures 3A, B displays that the time needed to restart the moving effector was higher in Mix condition. Accordingly, the analysis of the Stop Error trials detected a violation of the assumption of independence of the race model when selective inhibition was required in the Mix condition. On the contrary, it did not occur for non-selective conditions. By referring to the restart model, we hypothesized that during the non-selective conditions, the recruitment of only the first step was sufficient to stop the ongoing movement. Considering this step of the process to evolve according to the assumptions of the original race model (Logan and Cowan, 1984), we observed in these conditions that the MusRTs comply with independence assumption (Figures 4A, B, left panels). For these task conditions, we estimated a comparable SSRT that varies between 140 and 160 ms (see Table 2), which reliably match the ones estimated for single effector versions of SST (Jana et al., 2020). In the present data, we also observed that in the selective stopping of the Block condition, Stop Wrist and Stop Foot, the assumption of independence was on average respected (Figure 4A, middle and right panels), even though we detected a proportion of Non-Compliant trials. Likely, in these task conditions the foreknowledge of which effector to inhibit facilitated the restarting of the moving effector and we mainly detected inhibitory Stop Errors made during the first step. The smaller proportion of restarting errors and the lower duration of their MusRTs (see below and Figure 5) were not sufficient to violate the assumption of independence. According to this interpretation, we computed the SSRT for all these conditions and observed that they were not significantly different from those previously estimated in the non-selective conditions (see Table 2). On the contrary, in the Mix conditions, we detected a violation of the assumption of independence in both selective stop conditions (Figure 4B, middle and right panels). In this case the lack of foreknowledge of the effector to be inhibited required an effort to interpret the instruction delivered by the stop signal, and eventually, it increased the time needed for decoupling the two effectors. Since these error trials followed a previous stage of inhibition, they needed to be separated from the error trials used to test the assumption of the race model. Their longer MusRTs could lead to detection of a violation of the independence assumption that did not occur.

### 4.3. Compliant and Non-Compliant Stop Errors

Here we refer to a restart model to account for the violation of the independence assumption. In this framework, not all the selective Stop Errors occur because the Go process wins the race against the Stop process. Some errors occur in a second step of the process, when the two effectors need to be decoupled, allowing the restart of one of them. The Stop Error MusRT occurring for a wrong restarting of the moving effector should be excluded by the MusRT distribution used for testing the assumption of independence. Here we used the estimate of SSRT for each participant in non-selective Stop condition (see Table 2) as an estimate of the time needed for the Stop process to complete the general first step of inhibition. Then, we used each individual SSRT and average SSD to estimate the average end time of the Stop process (Stop End). For each selective Stop condition, this time point was used to separate the Compliant Error MusRTs, occurring during the first step (Stop Error MusRTs < Stop End), and the Non-Compliant Stop Errors, occurring during the second step (Stop Error MusRTs > Stop End) of inhibition. According to our hypothesis, we detected that in selective stop conditions, the percentage of Compliant MusRTs in the Stop Error trials were on average (*M* = 80 ± 2%) lower than the Stop End in the Block conditions (Figure 5B). This percentage further decreased (*M* = 69 ± 5%) in the Mix condition. The MusRTs of these Stop Error trials were significantly lower than in No Stop trials, confirming that they comply with the assumption of independence. We used the proportion of Compliant Stop Error trials to estimate the SSRT in the selective stop condition, and found them to be non-statistically different from those of the non-selective stop condition. All these results suggest that a two-step model reliably accounts for effector selective inhibition and provides a way to estimate the duration of the first step of inhibition. Violations of race model’s independence are common and occur independently of specific variables, e.g., different types of experimental manipulations, participants that are classified as being slow or fast, task designs that require selective or global inhibition with the involvement of simple or multiple effectors (Bissett et al., 2021). This violation occurs mainly for shorter SSDs, where a failure in triggering the Stop process is hypothesized (Logan and Cowan, 1984; Matzke et al., 2017). Accordingly, here we detected Non-Compliant Errors to occur for shorter SSDs (see Table 3). Even though this data seems to be in line with a failure in triggering hypothesis, a possible alternative explanation for Non-Compliant Stop Errors is that the errors occurred due to a failure in uncoupling the two effectors (Figure 5A). In other words, after a general inhibition took place, these trials started as correct trials, since the proper effector was restarted, and then turned into Stop Error trials because of a failure in uncoupling the two paired effectors. More in-depth analysis revealed that most of the Non-Compliant Errors were the trials where participants correctly started the moving effector, but the controlled effector to stop was still paired to the moving effector. In line with our restart hypothesis, in a small proportion of Non-Compliant Stop trials, we observed a failure in triggering the movement of the correct effector (Block: *M* = 16 ± 2%; Mix: *M* = 25 ± 5%). This outcome is in line with a failure in restarting rather than failure in stop triggering.

### 4.4. Implication for SST

**FIGURE 6:**
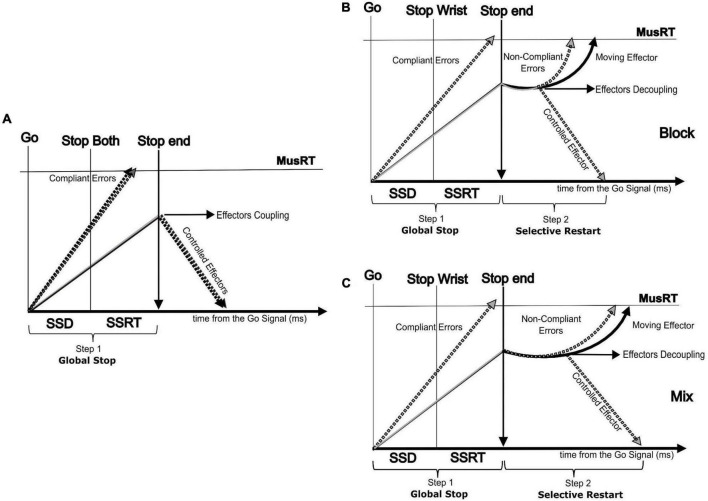
Two step (Restart) model showing the paired run of the two effectors followed by an effector decoupling process in the second step (selective restart). **(A)** Non-selective Stop version (Stop Both) showing the run of the two effectors followed by a global effector coupled inhibition process (first step); selective Stop version (only Stop Wrist shown) showing the run of the two effectors by an effector decoupling process in the second step (selective restart of only the foot) in the Block condition **(B)** and Mix condition **(C)**.

The present work fits the recent approach aimed at testing if the SST is a valid paradigm for studying motor inhibition not only when a single effector is involved in a movement but even when inhibition requires the selection of a single effector or a multi-component movement (Hannah and Aron, 2021). The assumptions of the horse race model used to account for the inhibition in the SST for single effectors sometimes are not achieved in selective versions of the task, especially for shorter SSDs (Boucher et al., 2007; Gulberti et al., 2014; Bissett et al., 2021). In these cases, it has been observed that the assumption of independence between the Go and the Stop processes is violated, i.e., the RTs of Stop Error trials are slower than No Stop trials (Bissett et al., 2021). Updated versions of the model, where the Go and the Stop processes interact with each other, have been developed to account for these detected violations (Boucher et al., 2007; Logan et al., 2014). Alternative models, still relying on a competition between the Go and the Stop processes are available to account for stimulus (Bissett and Logan, 2014; Giarrocco et al., 2021; Andujar et al., 2022) and response selective inhibition (de Jong et al., 1995; MacDonald et al., 2012; Xu et al., 2015; Wadsley et al., 2022). Among them, a two-step “restart” model (Figure 6) has been proposed for accounting for the selective response inhibition (de Jong et al., 1995; MacDonald et al., 2012; Xu et al., 2017; Wadsley et al., 2022).

According to this model, the selective inhibition follows an initial step where a general inhibition is applied on each possible motor output, both to the target effector (Aron and Verbruggen, 2008; MacDonald et al., 2012) and effectors completely independent from the ongoing task (Cai et al., 2012). This general inhibition pauses the rising of the Go process of the moving effectors toward the threshold (Wadsley et al., 2022). The consequence of this pausing is a cost that delays the MusRTs of the moving effectors (Aron and Verbruggen, 2008; MacDonald et al., 2012; Wadsley et al., 2022). Here we complement this model by showing that the cost of the pause is extended to selective Stop Error trials and that this cost would lead to a violation of the independence assumption. We hypothesize that the observed cost depends on the effort of the motor system in uncoupling the effectors functionally paired by the task instruction. In addition, we observed that this cost was context dependent since the probability to restart the proper effector depended on foreknowledge of which of the two effectors to stop. When it lacks, we observed an increased cost for interpreting the perceptual information and then engaging in the decoupling. On the contrary, the analysis of the No-Stop MusRT did not reveal a context effect that suggested a proactive inhibition (slower MusRT) in the condition with no foreknowledge of the effector to be inhibited, as detected in No-Stop RT with respect to the Go only RT (Mirabella, 2021).

A possible alternative approach to selective inhibition in Block conditions where the independence assumption was respected is provided by proactive inhibition, where the difficulty of the context or the outcome of the previous response influences the current one (Aron and Verbruggen, 2008; Bissett and Logan, 2014; Brunamonti et al., 2014; Genovesio and Ferraina, 2014; Mione et al., 2015). In this case, the foreknowledge of the stopping effector when the stop signal occurs should delay all the responses and then delay the RT of the moving effector as the SSRT (Aron and Verbruggen, 2008). This is not the case of the data presented in this work, since the SSRT in selective and non-selective stops were comparable and more in line with a reactive process as the restart hypothesis assumes (Table 1). We believe that in Block conditions, even if it was possible to predict the occurrence of the upcoming events, a proactive control could not be exercised since the unpredictable stop signal occurred during the motor preparation. Such events have been observed to elicit a general motor inhibition (Iacullo et al., 2020; Wadsley et al., 2022).

## 5. Conclusion

To summarize, the present findings support the view that even if inhibition is working effectively when selection is required, several intervening variables can interfere with the selection process. Here we suggest that the degree of difficulty in detecting the inhibitory Stop Signal influences the effectors decoupling leading to selective Stop Errors. By accounting for these errors, it will be possible to estimate the SSRT if the assumption of independence is violated. Overall, our results suggest that the application of the race model for estimating the SSRT in effector selective SST needs to account for a proportion of restart errors to increase its accuracy. Attention to such Stop Error trials will improve the efficiency of the SST as a useful tool in studying effector selective inhibition in clinical populations.

## Data availability statement

The raw data supporting the conclusions of this article will be made available by the authors, without undue reservation.

## Ethics statement

The studies involving human participants were reviewed and approved by Ethics Committee of Roma Tre University. The patients/participants provided their written informed consent to participate in this study.

## Author contributions

EB conceived the original idea. EB, PP, and SFe supervised the project. IM, VG, and EB conceived and performed the data analysis. EB and IM wrote the manuscript. All authors aided in interpreting the results reported in the final version of the manuscript.

## Footnotes

1
https://it.mathworks.com
